# Frost Bite

**Published:** 1873-01

**Authors:** 


### FROST BITE

Is the result of blood becoming so cold as not to circulate, and mortification and the death of the part takes place. When a limb on any part of the body is frozen it looses all sensibility and the skin becomes white; as soon as this is noticed, rub it gently with snow; the next best is ice water, for that is slightly warmer than the frozen part and thus changes the temperature by very slow degrees; keep it in cold water until the feeling returns and for two or three minutes later, then add a little warm water and in two or three minutes a little more, rubbing the part gently with the hand so as to promote the circulation.

If a person seems to be nearly frozen to death, remove all the clothing and cover the whole person, excepting the mouth and nose, in snow; if this cannot be had, use ice water, containing lumps of ice; after remaining a few moments, long enough to have some sensibility, take out the body and wipe it with cloths dipped in cold water until the muscles begin to relax, then remove to a cold bed, cover the body over and with the warm hands under the cover patiently rub the whole surface, for hours if necessary; two or three persons might be rubbing at the same time, in order to get up a circulation. If signs of life appear, give an injection of camphor water and put a few drops of spirits of camphor on the tongue. As soon as the person can notice things, give a teaspoonful of strong tea or coffee, and after a while give half a cup hot, at a time; not only parents, but all young persons ought to know these things. Two winters ago, a young gentleman advised a young lady, who was returning from skating with feet benumbed with cold, to put them in warm water as soon as she reached home; she did so to one foot, which had to be taken off.

